# Low-Temperature Sealing Material Database and Optimization Prediction Based on AI and Machine Learning

**DOI:** 10.3390/polym17091233

**Published:** 2025-04-30

**Authors:** Honghao Jia, Zhongxu Tai, Rui Lyu, Kousuke Ishikawa, Yixiao Sun, Jianting Cao, Dongying Ju

**Affiliations:** 1 Department of Information System, Saitama Institute of Technology, Fukaya 3690293, Japan; i2002ica@sit.ac.jp (H.J.); cao@sit.ac.jp (J.C.); 2 Tokyo Green Power Electric Research Institute Co., Ltd., Tokyo 1110022, Japan; taizx0224@gmail.com (Z.T.); lr518ever@gmail.com (R.L.); ishikawa@gpower.jp (K.I.); 3 School of Computer Science and Software Engineering, University of Science and Technology Liaoning, Anshan 114051, China; 202081203562@stu.ustl.edu.cn

**Keywords:** low-temperature sealing materials, machine learning, data mining, LLM-generated data, optimized design of materials

## Abstract

Optimization of low-temperature sealing materials is of great significance to improving low-temperature performance and durability. This study leverages DeepSeek-v3 (DS) and GPT-generated data and applies machine learning methods, including XGBoost and neural networks, to perform 3D prediction and analysis of key properties of low temperature sealing materials. Data expansion techniques were employed to enhance data quality and improve model prediction accuracy. Additionally, the study evaluates the applicability of AI-generated data in material performance prediction. The results demonstrate the effectiveness of machine learning in material optimization and provide valuable insights for future optimization strategies.

## 1. Introduction

Low-temperature sealing materials have garnered increasing attention as critical components in modern clean energy and industrial applications, owing to their essential role in maintaining system integrity under extreme conditions [1]. Their high performance, environmental compatibility [2] and versatility make them suitable for a wide range of uses, including cryogenic storage systems, aerospace engineering, liquefied gas transport, hydrogen fuel cells and other temperature-sensitive technologies [3]. As the global shift towards sustainable energy accelerates and the demand for efficient, low-emission systems grows, the development of reliable low-temperature sealing solutions becomes increasingly vital [4]. Therefore, the development of sealing rubbers that can withstand temperature changes within the range of low and high temperature environments, have high strength and reliable mechanical properties and chemical properties such as high electrical resistance and corrosion resistance, is receiving increasing attention [5].

### 1.1. Importance and Challenges of Low-Temperature Sealing Materials

As shown in Table 1, the following sealing materials sealing are primarily composed of rubber-based composites, designed to prevent gas leakage, withstand mechanical stress and maintain long-term stability under extreme operating conditions [6]. Low-temperature sealing materials serve multiple functions, including preventing leakage of contents, thus ensuring safety; withstanding extreme temperatures and chemical exposure, low-temperature operates at temperatures below −40 °C and are subject to acidic environments; maintaining insulation to prevent short circuits; providing mechanical strength and elasticity to accommodate temperature variations and structural deformations [7].

Despite their advantages, existing sealing materials face several challenges that impact low-temperature performance and durability: chemical degradation occurs, which shortens the material life [12]; extreme temperatures and mechanical instabilities lead to material fatigue [13]; excessive diffusion of gases stored in low-temperature sealing materials can reduce system efficiency and pose safety risks [14]; aging and long-term durability are concerned, as fluctuating environmental conditions accelerate material degradation; material cost and processing complexity, which hinder the large-scale production of high-performance sealing materials [15].

To overcome these limitations and enhance the reliability of low-temperature materials, it is essential to explore innovative materials and design strategies.

### 1.2. Optimization Strategies for Low-Temperature Sealing Materials

To address these challenges, researchers have proposed several optimization strategies to improve the durability and efficiency of low-temperature sealing materials:

- High-performance composite materials that enhance chemical resistance [16,17];
- Self-healing materials can extend the material life [18];
- Surface treatment and coating technologies, including plasma surface treatment and fluorinated coatings, to enhance chemical resistance and improve wettability [19].

Traditionally, the development of low-temperature sealing materials requires extensive physical testing to evaluate the material’s performance. This approach greatly limits the efficiency of material optimization.

Compared to general material design tasks, the design of low-temperature sealing materials is more challenging due to limited sample availability and the need to meet multiple performance objectives simultaneously.

### 1.3. Application of Machine Learning in Material Optimization

In recent years, machine learning (ML) has played an increasingly important role in materials science, significantly improving the efficiency of material design, optimization and performance prediction [20]. Among various techniques, supervised learning models such as random forest, XGBoost, and neural networks have demonstrated high accuracy in predicting material properties based on their compositions [21]. These models enable the prediction of new formulations prior to physical testing, thereby reducing experimental costs and enhancing research efficiency [22].

However, despite their potential, the application of machine learning in low-temperature sealing materials remains underexplored, with several critical research gaps:

- Existing studies are primarily based on experimental data, lacking validation for AI-generated data augmentation methods;
- Supervised learning is not optimized well enough for cryogenic sealing materials;
- No comparative studies have been conducted on the effectiveness of DS data (DeepSeek-v3-v3-v3) vs. GPT-generated data in material optimization.

A major challenge in applying machine learning to materials science is the limited availability of high-quality training data. To address this, researchers have adopted data augmentation and synthetic data generation techniques, including the following:

- Probabilistic interpolation—generating additional data points by estimating values between known experimental samples;
- Monte Carlo simulations—using statistical sampling methods to synthesize realistic material properties;
- Generative Adversarial Networks (GANs)—learning complex data distributions to generate synthetic yet highly realistic material datasets [23].

These techniques allow machine learning models to generalize more effectively and improve predictive performance, reducing dependence on costly physical experiments and accelerating material innovation.

This study proposes a small-sample multi-objective machine learning-based material optimization approach, which leverages artificial intelligence (AI) to predict the effects of a larger number and broader range of material formulations on sealing performance, thereby significantly accelerating the formulation design process [24].

By implementing these data mining and AI-generated optimization techniques, future low-temperature sealing materials are expected to achieve improved durability, sealing performance, chemical stability and environmental adaptability, ultimately accelerating the commercialization of low-temperature sealing materials [7].

### 1.4. Research Objectives and Contributions

The main objective of this study is to accelerate the design and optimization of low-temperature sealing materials by integrating experimental data, AI-generated synthetic data and advanced machine learning techniques:

- Construct a comprehensive Low-Temperature Sealing Material Database (LTSMD) that unifies material composition, physical properties and experimental performance metrics;
- Address key challenges in cryogenic sealing material design, such as limited sample availability and the need to balance multiple performance criteria (e.g., rebound rate, volume resistivity and acid resistance);
- Introduce data augmentation strategies, including probabilistic interpolation, ChatGPT4o4o-generated data and DeepSeek-v3-enhanced data, to expand the design space and improve prediction robustness;
- Apply and compare multiple machine learning models (e.g., XGBoost, MLPRegressor) to perform multi-objective prediction and uncover nonlinear relationships between formulation and performance;
- Clarify the differences in predictive behavior between DeepSeek-v3 and GPT-generated data, thereby providing insight into their respective contributions to materials modeling;
- Provide a validated, user-oriented tool for real-time querying, analysis and decision-making, enabling more efficient material screening and optimization.

Through these efforts, this research contributes to the literature by proposing a novel AI-driven framework for low-temperature sealing material optimization, offering a comparative analysis of data generation strategies and demonstrating the feasibility of augmenting small experimental datasets with AI-generated data for enhanced material innovation.

To address the challenges in material selection and performance optimization, this study establishes the Low-Temperature Sealing Material Database (LTSMD) and proposes a small-sample, multi-objective machine learning optimization method based on LLM-generated data. The approach systematically integrates experimental data, machine learning predictions and data augmentation techniques, providing comprehensive data support and decision-making tools for the design and optimization of low-temperature sealing materials.

## 2. Materials and Methods

This study employs a machine learning (ML)-based optimization approach for low-temperature sealing materials, involving the construction of a database, application of various data augmentation techniques and predictive analysis using XGBoost and neural networks (MLPRegressor). Through data mining, feature engineering, model optimization and 3D predictive analysis, a comprehensive material performance prediction framework has been developed.

### 2.1. Materials and Experiment

The main raw materials used in the experiments conducted in this study include the following: EBT-K9330M rubber, a product of Mitsui Chemicals, Inc.; PAO401 plasticizer, a product of Nippon Steel Corporation; Viscort#230 crosslinking aid, a product of Osaka Organic Chemical Industry Ltd.; SPHERON 5200 (GPF-grade) carbon black, a product of Cabot Japan K.K; calcium carbonate, a product of Shiraishi Calcium Kaisha, Ltd.; organic peroxides (PERHEXA-C/PERCUMYL-D), products of NOF Corporation; non-flex anti-aging agent, a product of Seiko Chemical Co., Ltd. The main equipment and instruments used in the experiments are as follows: compression set test—custom-made equipment; compression stress relaxation test—MCT-2150, manufactured by AND Corporation; resilience test—KY-1011S, manufactured by Xiamen Lianhe Zhonggong Instrument Co., Ltd.; low-temperature (−40 °C) resilience test refrigeration unit—RER-100, manufactured by REMACOM Corporation; hot and humid aging test equipment—CD-WY30 heater, manufactured by Zojirushi Corporation; impedance test—HT-800 MCP, manufactured by Nitto Seiko Analytech Corporation. The test standards followed in this study are as follows: EPDM compression set test—conducted in accordance with GB/T 7759.1-2015 (Chinese National Standard); EPDM compression stress relaxation test—conducted in accordance with GB/T 1685-2008 (Chinese National Standard); determination of resilience of vulcanized EPDM rubber—conducted in accordance with GB/T 1681-2009 (Chinese National Standard) [25,26,27]; hot and humid aging test method for vulcanized EPDM rubber—conducted in accordance with GB/T 15905-1995 (Chinese National Standard); determination of insulation resistivity of vulcanized EPDM rubber—conducted in accordance with GB/T 1692-2008 (Chinese National Standard); test method for liquid resistance of vulcanized or thermoplastic EPDM rubber—conducted in accordance with GB/T 1690-2010 (Chinese National Standard).

### 2.2. Data Collection

The data utilized in this study consists of two main sources: experimental data—real-world laboratory test results; AI-predicted data—generated using ChatGPT and DeepSeek-v3. Table 2 shows some sealing material data obtained from actual experiments. The examples in the table try to obtain more comprehensive data by maintaining other ratios unchanged and changing the carbon black content. Table 3 presents several AI-generated material formulations that meet the target criteria, along with their corresponding performance indicators.

### 2.3. Data Preprocessing and Augmentation

To ensure consistency, completeness and suitability of the experimental data for machine learning model training, systematic preprocessing and augmentation techniques were applied.

#### 2.3.1. Data Preprocessing

Data preprocessing is a critical step for enhancing model accuracy and stability. The following techniques were implemented to optimize input data quality:

- Column name standardization: removing extra spaces and replacing special characters to ensure format consistency;
- Missing data handling: missing values were filled using forward filling to maintain data continuity;
- Data normalization: to eliminate the impact of different variable scales, min-max scaling was applied using the following formula:

(1) $$x^{*}\;=\;\frac{x\;-\;min(x)}{max(x)\;-\;min(x)}$$

where $x^{*}$ represents the normalized data, *x* is the original data and $\min\left(x\right)$ and $\max\left(x\right)$ are the minimum and maximum values of the dataset, respectively.

Data cleaning: removing redundant or erroneous data to enhance quality and prevent the influence of outliers on model training [28].

#### 2.3.2. Data Augmentation

To improve the model’s generalization capability and prediction accuracy, data augmentation techniques were employed. These techniques expanded the training dataset through synthetic sample generation, interpolation and probabilistic sampling:

Parameter variation and interpolation: by slightly adjusting existing data parameters and applying linear or nonlinear interpolation, new material compositions were generated, enriching dataset diversity.

Synthetic sample generation: additional training data were created by randomly generating new samples based on the statistical distribution of the original data, improving the model’s adaptability to different material formulations.

Random perturbation: small-scale random noise was added to existing data to introduce controlled variability, simulating experimental errors and enhancing model robustness.

Interpolation-based data augmentation generates additional training samples by estimating intermediate values between known material formulations [29].

This method ensures continuous distribution of material properties in the dataset, improving model generalization.

To introduce controlled variability and simulate real-world deviations, Gaussian noise perturbation was applied. This method enhances model robustness, making it adaptable to minor variations in experimental conditions.

Additionally, Monte Carlo-based synthetic data generation was employed using statistical sampling techniques. By generating synthetic data points that mimic real experimental results, this technique effectively expands the dataset size without requiring additional physical experiments.

To further improve computational efficiency, Principal Component Analysis (PCA) was applied for feature reduction.

### 2.4. Database Construction (LTSMD)

This study establishes the Low-Temperature Sealing Material Database (LTSMD) to store material composition, physical properties and experimental test results. The database is named Sealing Material Design and Performance Retrieval Software (SM-D&P) and is designed as follows:

#### 2.4.1. Database Architecture and Data Import

The system uses SQLite as the backend database for efficient data management and retrieval. The database organizes materials hierarchically based on material type, model and associated data tables, such as composition, physical properties and performance test results. Each table follows a standardized naming format, e.g., SyntheticRubber_EBT_Composition.

Data from Excel files are automatically parsed, cleaned and imported into the database using custom scripts, ensuring completeness and consistency of material information. This allows for unified data handling and enables downstream machine learning applications.

#### 2.4.2. Database Interface Overview

**Figure 1:**
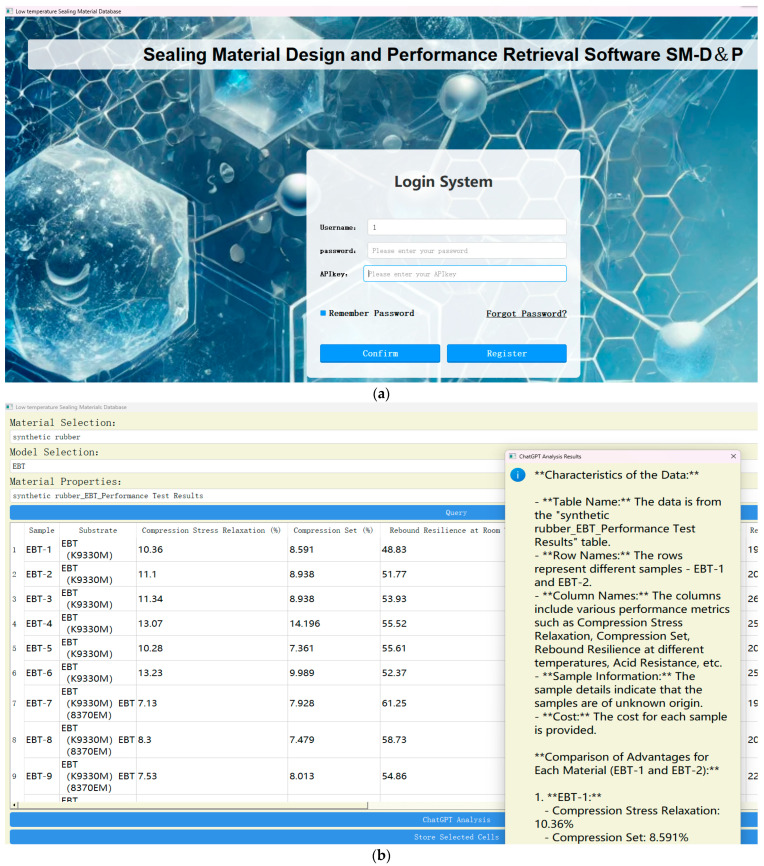
Login interface of database (**a**) and main interface of database (**b**).

As shown in Figure 1, LTSMD consists of the following interface components:

Cover window: where users click “Enter” to access the login interface.

Login interface: for user login, registration and API key management.

Main interface: supports material selection, query execution and intelligent analysis.

#### 2.4.3. Database Functionality and Data Structure

The LTSMD database includes the following:

- Material composition table: stores chemical composition and additive ratios;
- Physical properties table: includes properties such as density, hardness and modulus;
- Performance testing table: records experimental results of rebound resilience, volume resistivity and acid resistance.

Key functionalities include query and analysis of different materials (e.g., synthetic rubber, silicone rubber and UV adhesives); simultaneous display of formulation and performance for specific materials; data selection and ChatGPT analysis tool for evaluating performance, mass production feasibility and low-temperature suitability.

To increase data coverage and predictive robustness, the database integrates data expansion techniques, including interpolation methods; ChatGPT-generated synthetic data; DeepSeek-v3-based data augmentation.

These augmentations help mitigate the limitations of limited experimental data and enhance the quality of machine learning model predictions.

### 2.5. Machine Learning Methods

This study employs XGBoost and neural networks (MLPRegressor) for predicting the performance of low-temperature sealing materials, integrating hyperparameter optimization to enhance model accuracy.

XGBoost (gradient boosting decision trees) suitable for nonlinear data, capable of handling complex relationships between material composition and performance.

Hyperparameter optimization utilizes grid search to fine-tune learning rate, tree depth and L1 regularization parameters.

Feature importance analysis identifies the most influential components affecting material performance.

K-fold cross-validation (K = 5) enhances model stability and generalization ability.

Neural network (MLPRegressor) multilayer perceptron (MLP) regression is applied to capture complex nonlinear patterns in high-dimensional data. Network architecture: Input Layer—2~3 hidden layers—ReLU activation function—Output Layer. Adam optimizer is employed for gradient updates, preventing local optima traps. Early stopping is applied to prevent overfitting and improve generalization ability.

Mean Squared Error (MSE), Root Mean Squared Error (RMSE) and Coefficient of Determination (R²) are used to evaluate model performance. An 80–20% train-test split is adopted to assess the model’s generalization capability. XGBoost-based prediction of material performance handles nonlinear relationships between material components and performance metrics. Hyperparameter optimization (e.g., learning rate, tree depth and L1 regularization) improves predictive accuracy.

(2) $$\hat{y}\;=\;\sum \alpha_{m}\cdot f_{m}(x)$$

Feature importance analysis helps identify key material components affecting performance. MLP regression is used to learn complex high-dimensional data patterns.

**Figure 2:**
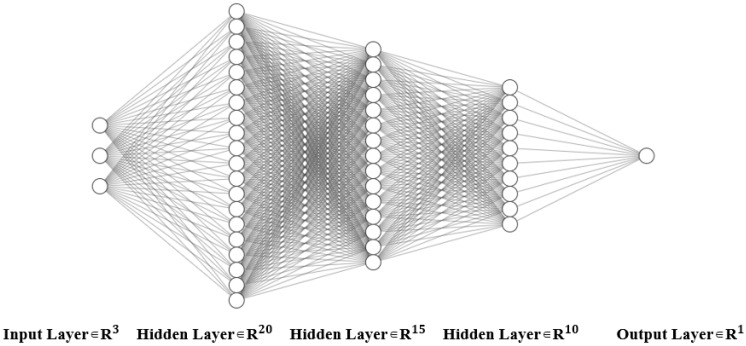
Neural network architecture example.

Network architecture (take Figure 2 as an example): 

Input Layer: 4 neurons (softener, carbon black, crosslinking agent and calcium carbonate).

Hidden Layers:

First hidden layer: 20 neurons (ReLU activation).

Second hidden layer: 15 neurons (ReLU activation).

Third hidden layer: 10 neurons (ReLU activation).

Output Layer: 1 neuron (predicting rebound resilience or volume resistivity or acid resistance).

Optimization Strategy:

Adam optimizer (learning rate = 0.001).

L2 regularization (α = 0.0001) to prevent overfitting and enable automatic feature selection.

Maximum iterations: 50,000 (for a 3-variable neural network example).

Cross-validation is performed to determine the optimal network architecture and avoid overfitting.

In this study, we employed single-output neural network architecture for each target variable independently. Specifically, for each performance indicator (i.e., rebound rate, log resistivity and acid resistance), a separate Multi-Layer Perceptron (MLP) Regressor was constructed and trained. Each model was designed to map the relationship between material composition variables and a single target response.

The decision to employ separate neural networks for each target variable instead of a multi-output model was based on the following considerations:

- Independent optimization of each target: training separate models ensures that each performance indicator is optimized independently, avoiding potential interference between different targets;
- Better convergence and stability: multi-output models may introduce additional complexity, leading to slower convergence and possible suboptimal solutions;
- Flexibility in feature selection: each target variable may have different key influencing factors and using independent networks allows us to optimize the feature selection and hyperparameters accordingly.

Neural network computation and training process

For each layer l, the linear transformation and activation function output are computed as follows:

(3) $$z^{\left(l\right)}\;=\;W^{\left(l\right)}a^{\left(l-1\right)}\;+\;b^{\left(l\right)}$$

(4) $$a^{\left(l\right)}\;=\;f\left(z^{\left(l\right)}\right)$$

where $W^{\left(l\right)}$ is the weight matrix (determined by the number of neurons in the current and previous layers); $b^{\left(l\right)}$ is the bias term (column vector); $a^{\left(l-1\right)}$ is the activation output from the previous layer; *f* (·) is the activation function (ReLU).

The hidden layers utilize the ReLU activation function:

(5) $$f(z)\;=\;\max\left(0,z\right)$$

For regression problems, the output layer typically does not use an activation function:

(6) $$y_{\mathrm{pred}}\;=\;W^{\left(\mathrm{out}\right)}a^{\left(L\right)}\;+\;b^{\left(\mathrm{out}\right)}$$

where $y_{\mathrm{pred}}$ represents the predicted rebound resilience or volume resistivity.

The loss function is Mean Squared Error (MSE):

(7) $$L\;=\;\frac{1}{m}\sum_{i=1}^{m}{\left(y_{\mathrm{pred}}^{\left(i\right)}\;-\;y_{\mathrm{true}}^{\left(i\right)}\right)}^{2}$$

L2 Regularization (Weight Decay):

(8) $$L_{reg}\;=\;\frac{\alpha }{2}\sum_{l=1}^{L}{\left|\left|W^{\left(l\right)}\right|\right|}^{2}$$

(9) $$L_{total}\;=\;L\;+\;L_{reg}$$

where α = 0.0001 controls the regularization strength.

Backpropagation algorithm: Error gradient computation from the output layer:

(10) $$\delta^{\left(L\right)}\;=\;\frac{\partial L}{\partial z^{\left(L\right)}}\;=\;\left(a^{\left(L\right)}\;-\;y\right)$$

For hidden layers:

(11) $$\delta^{\left(l\right)}\;=\;{\left(W^{\left(l+1\right)}\right)}^{T}\delta^{\left(l+1\right)}\cdot f^{\prime }\left(z^{\left(l\right)}\right)$$

where $f^{\prime }\left(z\right)$ is the derivative of the ReLU activation function.

We use the Adam optimizer for parameter update:

(12) $$W^{\left(l\right)}\;=\;W^{\left(l\right)}\;-\;\eta \cdot \frac{\partial L_{total}}{\partial W^{\left(l\right)}}$$

(13) $$b^{\left(l\right)}\;=\;b^{\left(l\right)}\;-\;\eta \cdot \frac{\partial L_{total}}{\partial b^{\left(l\right)}}$$

where *η* = 0.001 (learning rate).

### 2.6. Data Augmentation and Expansion Methods

Due to the limited availability of experimental data, this study employs multiple data augmentation techniques to enhance the generalization ability of the machine learning models.

#### 2.6.1. Interpolation Method

To address the limitations associated with the small quantity of experimental data, we adopted a hybrid data expansion strategy combining linear interpolation, LLM-based synthetic data generation and Monte Carlo sampling.

The application of linear interpolation is justified by the assumption that the material performance varies smoothly within small perturbations of the formulation space, particularly in quasi-linear ranges of composition-response behavior (e.g., softener vs. rebound rate). This assumption is grounded in empirical rheological and crosslinking theory, where small changes in constituent ratios result in proportionally gradual variations in mechanical or electrical properties. To minimize artifacts, interpolation was only applied between experimentally verified samples with similar base matrices (e.g., EBT-based EPDM). In sparse regions of the data space, interpolation was not applied to avoid introducing unrealistic gradients.

Regarding LLM-based data generation, we used prompt-based synthesis on pre-trained large language models (LLMs), specifically ChatGPT, without fine-tuning. Prompts were crafted using domain-specific patterns derived from experimental composition–property relationships. The responses were filtered based on physical feasibility constraints (e.g., phr balance, total additive limit and density range).

The numerical distributions of AI-generated properties (e.g., rebound rate and resistivity) closely match the empirical ranges observed in actual experiments (Figure S1, Supplementary Information). AI-generated formulations consistently follow known physical trends, such as increasing softener content enhancing elasticity or increasing carbon black reducing resistivity. These results demonstrate that, although not physically measured, the LLM-generated data maintains numerical consistency, trend alignment and predictive utility, thereby supporting their inclusion in data-driven material optimization.

To fill in missing experimental data and smooth the variation between variables, this study applies linear interpolation (LI). To introduce controlled variations and simulate potential deviations in material properties that may occur during manufacturing and testing, this study employs Gaussian Noise Perturbation (GNP):

(14) $$x_{perturbed}\;=\;x\;+\;\epsilon ,\;\epsilon \sim N\left(0,\sigma^{2}\right)$$

where ϵ follows a normal distribution with a mean of 0 and variance $\sigma^{2}$. This technique enhances the model’s robustness by simulating real-world experimental uncertainties and manufacturing tolerances.

To further expand the dataset without additional physical experiments, this study employs Monte Carlo-based synthetic data generation, a statistical sampling method that generates new material compositions and performance data:

(15) $$x_{synthetic}\sim N\left(\mu ,\sigma^{2}\right)$$

where *μ* represents the mean value of the dataset and $\sigma^{2}$ represents the variance. This technique generates realistic synthetic data that follows the statistical properties of the original dataset, thereby increasing the diversity of training samples.

To improve computational efficiency, this study applies Principal Component Analysis (PCA) for feature reduction while preserving the most informative components of the data. The application of PCA helps to eliminate redundant information, enhance computational efficiency and reduce the risk of overfitting. After data expansion and augmentation, the enhanced dataset is used to train supervised learning models such as random forest and neural networks, enabling more accurate predictions of new material properties. To continuously refine the predictive models, the study monitors key performance indicators such as Mean Absolute Error (MAE):

(16) $$MAE\;=\;\frac{1}{n}\sum_{i=1}^{n}\left|y_{i}\;-\;\hat{y_{i}}\right|$$

#### 2.6.2. Deep Learning-Based Data Augmentation

This study generates new data points based on the statistical distribution of the existing dataset:

(17) $$X^{\prime }\;=\;X\;+\;\epsilon \cdot X_{max}\;-\;X_{min}$$

where $X^{\prime }$ is the newly generated data, X is the original dataset, *ϵ* is a random perturbation factor sampled from a uniform distribution: (*ϵ*∼*U* (−0.1, 0.1))

Randomly sample from existing data to randomly select experimental samples while ensuring data validity.

Add controlled perturbations to introduce small changes (≤10%) in numerical features to avoid overfitting to known data.

To further improve the dataset and ensure that newly generated data adhere to physical constraints and align with the real material properties, a Variational Autoencoder (VAE) is employed to generate diverse synthetic material samples resembling real-world experimental data. The VAE learns the latent structure of the dataset and samples from this distribution to create new data points. This approach ensures that the generated data follows the underlying probabilistic distribution of real material compositions and properties.

A Generative Adversarial Network (GAN) is utilized to learn the data distribution and generate synthetic samples that closely resemble real experimental data. GANs operate by training two competing neural networks:

- Generator: produces synthetic data samples;
- Discriminator: differentiates between real and generated samples.

Through this adversarial training process, GANs generate highly realistic synthetic datasets, effectively augmenting the training set and enhancing the model’s generalization performance.

**Figure 3:**
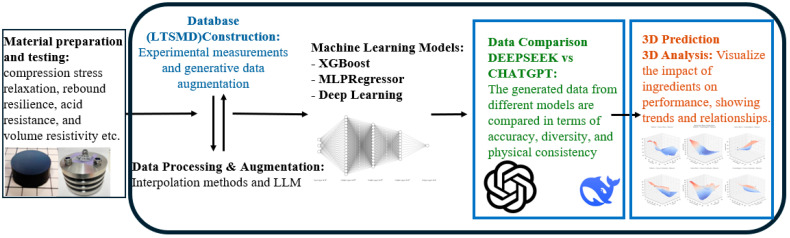
LTSMD database construction and ML-driven performance prediction framework.

Matplotlib and seaborn are used to generate 3D prediction curves. 3D visualization analysis is performed on different material ratios (softener, carbon black, calcium carbonate, etc.) to observe their effects on rebound rate, resistance rate and acid resistance. The reliability of different data sources is evaluated through gap comparison analysis (DS vs. GPT data). The research process framework is shown in the Figure 3.

## 3. Experiments and Results

Prediction of four variables and three target properties variable selection:

- Softener: affects elasticity;
- Carbon black: influences electrical conductivity;
- Crosslinking co-agent: impacts crosslink density;
- Calcium carbonate: affects acid resistance.

Target Properties:

- Low-temperature rebound: reflects the material’s ability to recover elasticity at extremely low temperature.
- Volume resistivity: measures the material’s electrical conductivity.
- Acid resistance: indicates the material’s reaction when exposed to an acidic environment.

Performance requirements: rebound rate at −40 °C should not be lower than 30%; volume resistivity should exceed 10^13^ Ω·cm; acid resistance should not exceed 0.12%.

### 3.1. Forecast Result Analysis

**Figure 4:**
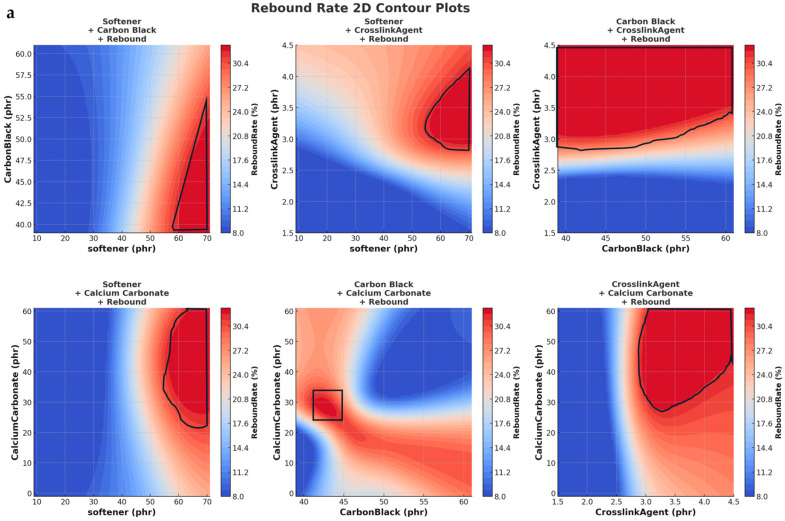

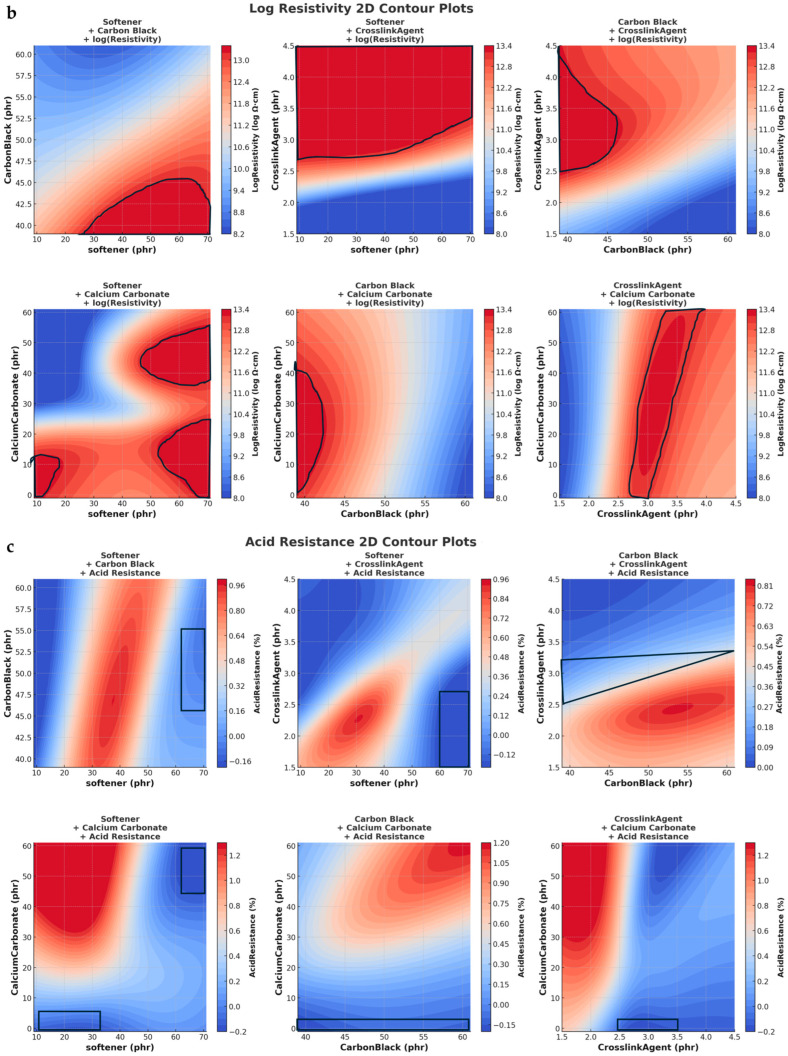
Rebound rate (**a**), resistivity (**b**) and acid resistance (**c**) of DeepSeek-v3 data prediction results.

**Figure 5:**
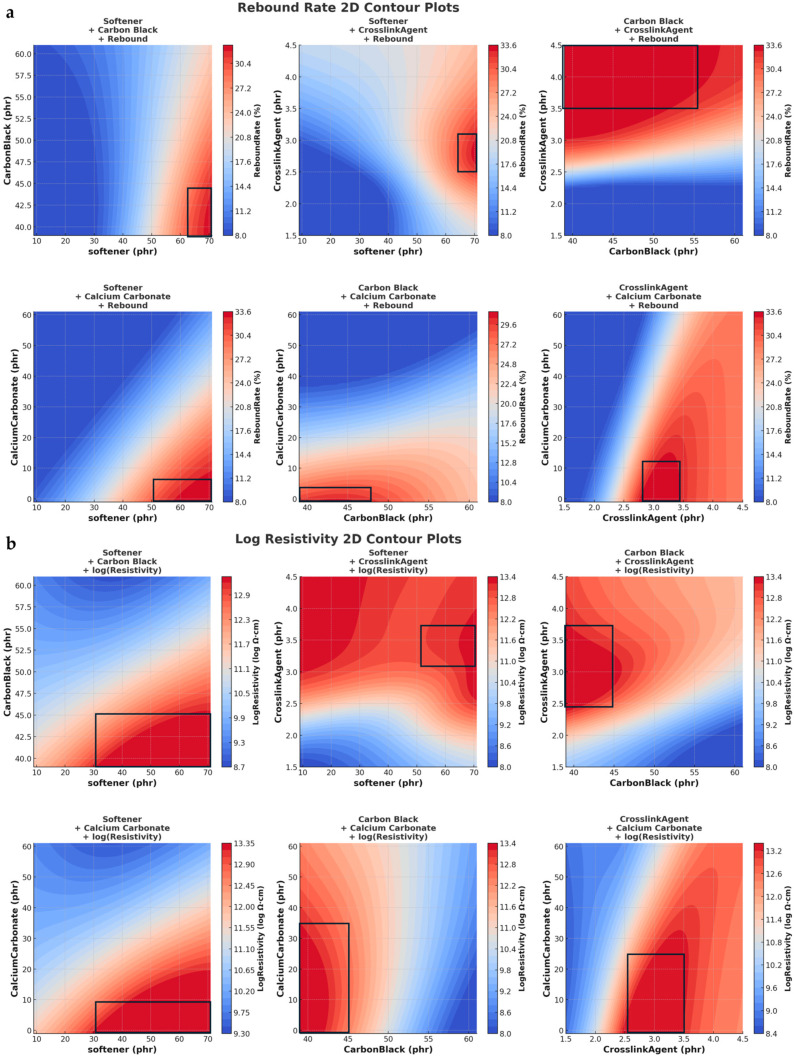

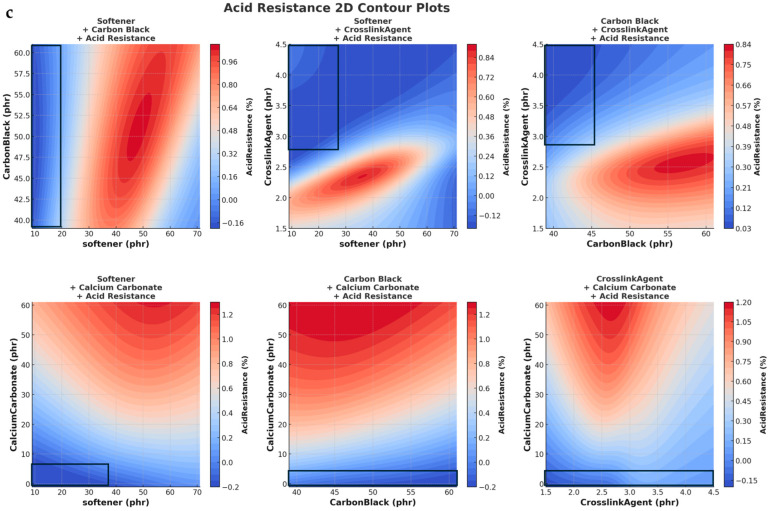
Rebound rate (**a**), resistivity (**b**) and acid resistance (**c**) of GPT data prediction results.

As shown in Table 4, which is derived from the valid prediction ranges observed in Figure 4 and Figure 5, DeepSeek-v3 predictions consistently indicate a narrower valid prediction range for crosslinking co-agent content (3.0–3.5 phr), compared to the broader range predicted by ChatGPT (2.75–3.25 phr or even 2.5–4.5 phr depending on the target property). This narrower range suggests a more targeted and stable optimization strategy, especially in achieving consistent mechanical bonding.

The crosslinking co-agent directly affects the formation of the three-dimensional network structure within the rubber matrix, which governs elasticity, durability and chemical resistance. A narrower and more focused range reflects the model’s identification of an optimal crosslink density required to maintain performance under low-temperature conditions.

In contrast, ChatGPT’s broader prediction intervals may allow for greater formulation flexibility but at the potential cost of increased variability in material behavior. This indicates that DeepSeek-v3 is better suited for precision-oriented design tasks where mechanical consistency and reliability are critical.

Therefore, these results imply that optimization strategies, particularly for parameters such as rebound rate and resistivity, should focus more on refining and controlling the crosslinking co-agent within a specific, narrow range to achieve stable and repeatable performance.

**Figure 6:**
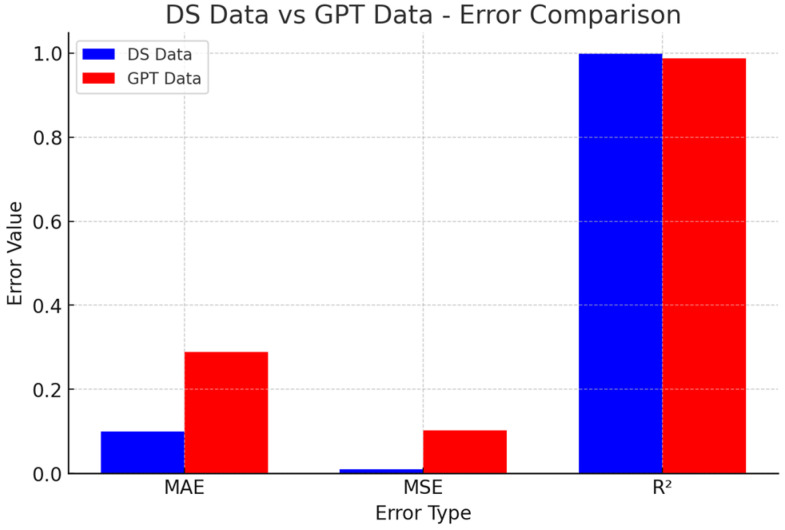
Error comparison histogram.

As shown in Figure 6, DeepSeek-v3 data: lower prediction errors, making it more reliable for capturing overall material behavior; better suited for general trend modeling, ensuring stable and consistent predictions; may be less responsive to complex nonlinear variations, potentially limiting flexibility in capturing intricate interactions.

CHATGPT data: more adaptive to highly nonlinear interactions, which may be beneficial for exploring complex material behaviors. This allows for greater flexibility in parameter adjustments, making it more useful for customized material design. There is a higher risk of overfitting, meaning that the model may fit noise rather than true trends, reducing generalizability in some cases.

### 3.2. Key 3D Prediction Analysis

**Figure 7:**
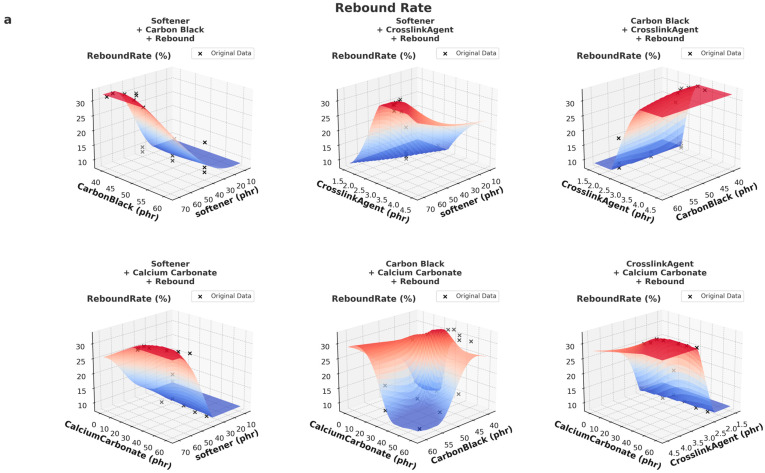

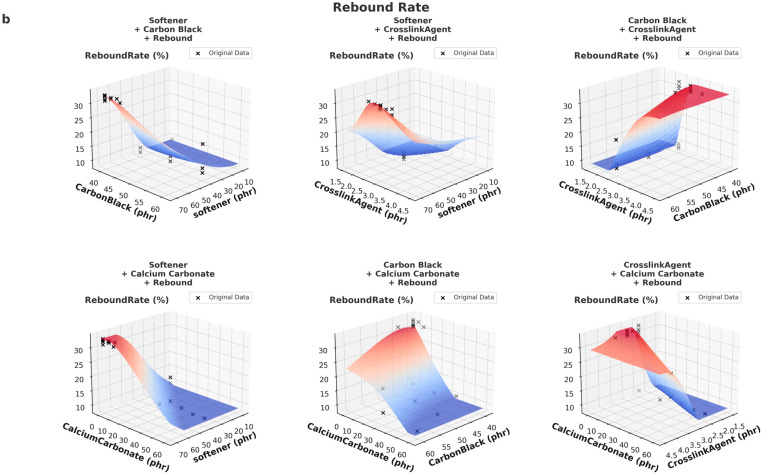
Comparison of 3D prediction of rebound rate of DeepSeek-v3 data (**a**) and GPT data (**b**).

As illustrated in Figure 7, both the DeepSeek-v3- and GPT-based models show a consistent increase in rebound rate with higher softener content and a mild decrease with increasing carbon black. These trends align well with the physical understanding of material behavior—softener improves elasticity, whereas excessive filler (carbon black) may reduce rebound efficiency due to increased stiffness.

The similarity in predicted surfaces across both models can be attributed to the fact that the underlying physical mechanisms are strongly represented in the training data. Despite differences in the source (DS vs. GPT), both datasets capture essential trends such as the positive contribution of softeners and the nonlinear influence of crosslinking agents.

**Figure 8:**
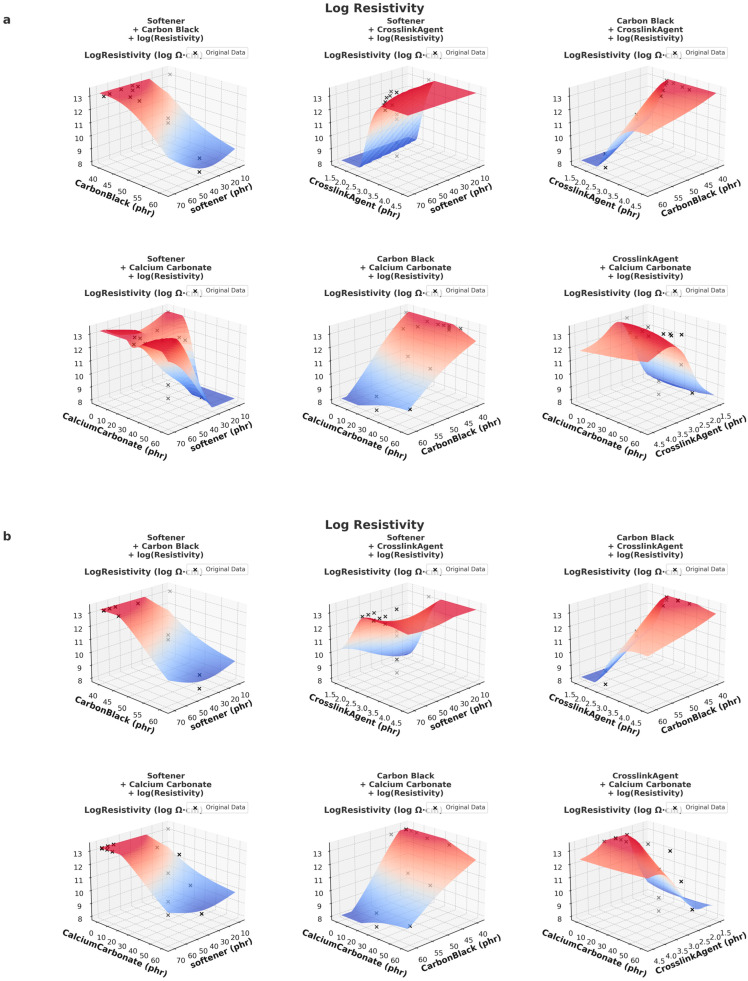
Comparison of 3D prediction of resistivity of DeepSeek-v3 data (**a**) and GPT data (**b**).

Moreover, both models were trained using similar supervised learning frameworks (XGBoost and MLP), which are effective at capturing dominant relationships even from partially synthetic datasets. This convergence in learning outcome indicates that both datasets contain sufficient and coherent representations of the material–performance relationships, thus leading to similar overall prediction surfaces. The following is an analysis of the trends presented in Figure 8.

DeepSeek-v3 data (a): the prediction surface is smoother, with a more gradual transition in lower resistivity regions; this suggests that the DS model has captured a stable and well-generalized trend, ensuring reliable interpolation across different material compositions.

GPT data (b): the prediction surface shows more local fluctuations, especially in the high resistivity region; this could be due to differences in numerical distribution within the GPT training dataset, leading to higher sensitivity to specific data points and potentially introducing more variation in resistivity predictions.

**Figure 9:**
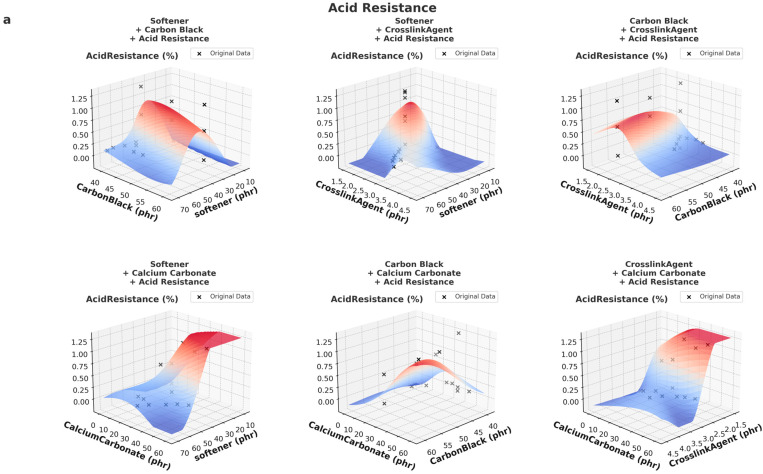

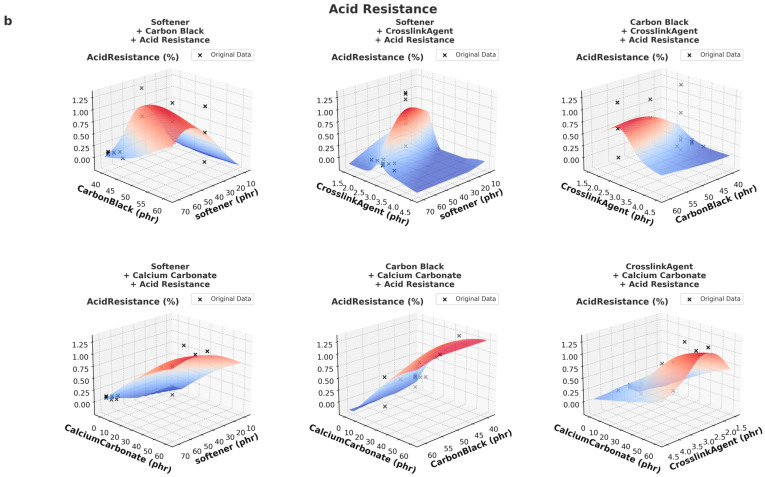
Comparison of 3D prediction of acid resistance of DeepSeek-v3 data (**a**) and GPT data (**b**).

As shown in Figure 9, DeepSeek-v3-based predictions exhibit smoother and more consistent trends in acid resistance and resistivity across different material formulations. This can be fundamentally attributed to the data generation mechanism of DeepSeek-v3, which is based on probabilistic interpolation and domain-specific modeling grounded in physical relationships. In this framework, new data points are generated by interpolating between known experimental results, ensuring numerical continuity, bounded variance and physically meaningful trends. Consequently, DeepSeek-v3 data presents a low-noise and well-structured dataset, enabling machine learning models to learn smooth, globally coherent patterns.

In contrast, GPT-generated data is produced through autoregressive token-by-token generation within a language model framework that lacks explicit numerical or physical constraints. While GPT can generate plausible numbers in context, it does not guarantee smooth gradients or monotonic transitions across numerical variables. This often leads to locally inconsistent data, especially in high-dimensional formulation spaces where relationships are complex and unstructured. Additionally, since GPT is not inherently trained on physical principles or interpolation structures, it may introduce artificial sharp changes or numerical noise, resulting in prediction surfaces with localized fluctuations or discontinuities.

These fundamental differences in data generation explain why DeepSeek-v3 models are more stable and predictable in response to parametric changes, whereas GPT models may be more exploratory but require additional regularization or integration with experimental constraints to achieve comparable reliability.

### 3.3. Data Relationship Distribution Diagram Analysis

**Figure 10:**
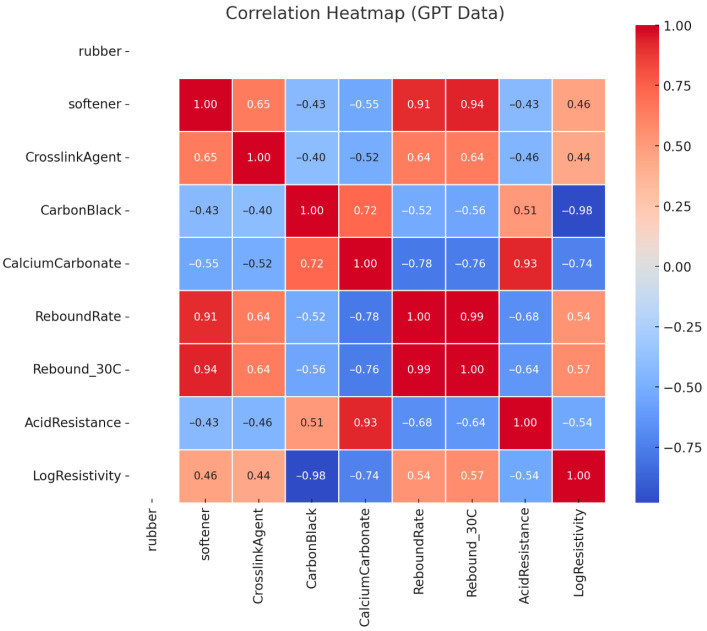
Comprehensive parameter relationship heat map.

The heat map presented in Figure 10 illustrates the interrelationships among input variables and key performance indicators, offering valuable insights for multi-objective formulation optimization. Notably, softener content exhibits a strong positive correlation with rebound resilience (r ≥ 0.90), confirming its critical role in enhancing material elasticity at low temperatures. However, it also shows a negative correlation with volume resistivity, indicating a trade-off between mechanical flexibility and electrical insulation performance.

Carbon black demonstrates a strong negative correlation with resistivity (r ≤ −0.90), highlighting its effectiveness in enhancing electrical conductivity. At the same time, excessive carbon black may lead to a slight reduction in rebound resilience, suggesting that its dosage must be carefully optimized to balance electrical and mechanical performance.

Calcium carbonate shows a positive correlation with acid resistance values, which, given that acid resistance in this context is measured by mass gain, actually implies a decrease in true acid resistance. This counterintuitive result is particularly important for chemical durability optimization, as it highlights the need to interpret correlated metrics carefully.

These findings support the development of informed optimization strategies. For instance, moderate-to-high softener levels may be prioritized to ensure sufficient elasticity; meanwhile, carbon black and calcium carbonate contents must be fine-tuned to balance resistivity and acid resistance. Furthermore, the observed cross-dependencies between parameters underscore the importance of constrained optimization approaches that account for multi-variable interactions.

**Figure 11:**
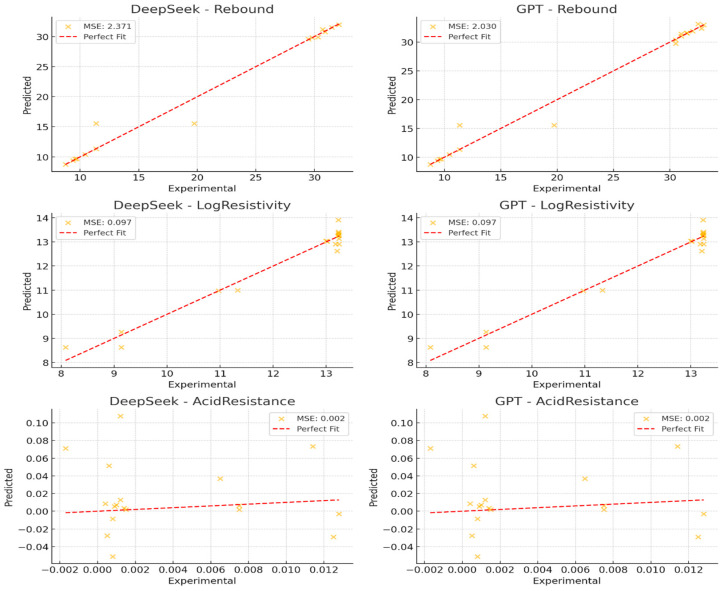
Scatter plot of experimental data vs. predicted data.

As shown in Figure 11, tfigurehe red dashed line represents an ideal fit where predicted values = experimental values. The closer the points are to this line, the lower the prediction error, indicating a well-generalized model.

DeepSeek-v3 data (left column): prediction values are more concentrated, with the point cloud aligned closely along the red dashed line, indicating low overall error. However, some high-value points appear underestimated, suggesting slight underfitting. This means the model may not fully capture extreme variations, leading to a smoother and more stable prediction trend but at the cost of less sensitivity to high-value deviations.

GPT data (right column): prediction values are more dispersed, indicating the model is more responsive to nonlinear relationships. However, some experimental values show significant overfitting, visible as high-error points deviating from the red dashed line. This means that GPT is capturing finer details but may also be overfitting noise or minor fluctuations in the data, reducing its robustness for generalization.

## 4. Discussion

This study focuses on data augmentation using machine learning methods (specifically XGBoost and MLPRegressor) to optimize low-temperature sealing materials. In this section, we discuss the applicability of these machine learning models, the impact of data characteristics on model performance, and future directions for improvement.

### 4.1. Applicability of Machine Learning Models

XGBoost demonstrates strong generalization capabilities, making it well-suited for capturing complex nonlinear relationships in material properties.

In this study, XGBoost effectively predicted rebound rate and log resistivity, but its prediction error for acid resistance was relatively high.

Neural networks can capture more complex features, but they are prone to overfitting with limited data.

On DeepSeek-v3 data, MLPRegressor effectively captured global trends, whereas on GPT data, local fluctuations led to higher errors.

Machine learning methods, such as MLPRegressor, perform better when dealing with a larger number of input variables.

### 4.2. Impact of Data Characteristics on Model Prediction

To evaluate the consistency between model-derived variable correlations and actual material behavior, a representative set of experimental samples (EBT-6, EBT-7, EBT-9, EBT-11, and EBT-16) were analyzed. These samples span a broad range of key compositional parameters, including softener, carbon black and calcium carbonate, and allow validation of predicted trends in rebound rate, volume resistivity and acid resistance.

The model predicted a positive correlation between softener content and rebound performance, which is supported by EBT-7 and EBT-6. Although EBT-7 contains only 13 phr of softener, it shows a high rebound rate of 61.25% at room temperature. However, this result is largely attributed to its low carbon black and calcium carbonate contents, illustrating the non-linear interaction effects. In contrast, EBT-16, with 40 phr softener but higher filler content (60 phr CaCO_3_), shows a lower rebound rate (48.63%), confirming the complex, non-monotonic relationships captured by the model.

In terms of electrical properties, EBT-6 and EBT-11 exhibit significant increases in volume resistivity (2.11 × 10^13^ Ω·cm and 1.43 × 10^13^ Ω·cm, respectively) compared to EBT-7 (8.9 × 10^7^ Ω·cm), despite relatively similar levels of carbon black. This agrees with the model’s prediction that combinations of lower filler loading and absence of calcium carbonate led to higher insulation capacity.

Regarding acid resistance, EBT-16 demonstrates the highest acid mass gain (0.0128%), consistent with its highest calcium carbonate loading (60 phr) and confirming the model’s expectation that increased filler dosage negatively impacts chemical durability.

Overall, these findings show that the machine learning models, despite being partially trained on AI-generated data, successfully captured both linear and non-linear multi-variable interactions that are reflected in real-world experimental trends.

### 4.3. Future Directions for Improvement

(1) Improving data quality and enhancing model robustness

Utilize Generative Adversarial Networks (GANs) or Variational Autoencoders (VAE) to generate more realistic synthetic data, reducing the overfitting risks in GPT-generated data.

Apply transfer learning, leveraging knowledge from other material optimization studies to enhance model generalization.

(2) Exploring more advanced deep learning architectures

Implement transformer-based architectures for sequence modeling, improving the model’s ability to learn complex material properties.

Utilize Graph Neural Networks (GNNs) to analyze microscopic material structures, integrating molecular-level information for more in-depth predictions.

(3) Optimizing feature engineering and variable selection

Apply automatic feature selection techniques such as LASSO regression or SHAP analysis to eliminate irrelevant variables and improve model efficiency.

Integrate physical modeling approaches, such as Finite Element Analysis (FEA), to combine experimental and simulation data, improving prediction accuracy.

### 4.4. Summary of Discussion

XGBoost is effective for global trend modeling, while MLPRegressor excels at capturing localized features.

Data quality determines model accuracy:

DS data is more suitable for stable, global trend analysis.

GPT data captures more complex features but requires optimization to mitigate overfitting risks.

Variable correlation analysis helps refine feature selection, improving model interpretability.

Future research should explore:

Deep learning architectures (Transformers, GNNs)

Data augmentation techniques (GANs, VAEs)

Hybrid approaches integrating physical modeling (FEA)

By improving machine learning techniques and enhancing data quality, we can further optimize the performance prediction of low-temperature sealing materials, ensuring more accurate and reliable results for industrial applications.

## 5. Conclusions

This study utilized specific experimental data to establish a Low-Temperature Sealing Material Database primarily focused on rubber-based materials. We utilized DeepSeek-v3 (DS) data and GPT-generated data, employing XGBoost and neural networks (MLPRegressor) to predict key properties of low-temperature sealing materials, including rebound rate, volume resistivity and acid resistance. A comparative analysis of DS-generated data and GPT-generated data in low-temperature sealing material optimization demonstrated that DS data is more suitable for global trend modeling, effectively capturing the steady-state variations in experimental data, whereas GPT data is more sensitive to local feature learning but may be prone to overfitting risks. A multi-source data fusion approach was developed for optimizing low-temperature sealing materials, integrating experimental data with AI-generated data (DS/GPT) to enhance data coverage and improve model generalization capabilities. By employing probabilistic interpolation, ChatGPT-generated data and DeepSeek-v3 data augmentation, this approach effectively addresses data scarcity issues, providing a novel pathway for data-driven material optimization. The study further optimized machine learning models for predicting low-temperature sealing material properties, revealing that XGBoost is well-suited for global modeling, while MLPRegressor demonstrates superior performance in capturing localized features. Through feature importance analysis, the study identified the nonlinear effects of key variables such as softener, carbon black and crosslinking agent on material performance, accompanied by 3D visualization analyses to enhance model interpretability.

Additionally, a 3D prediction-based error comparison method was proposed, utilizing 3D surface visualizations of rebound rate, volume resistivity and acid resistance to intuitively compare the impact of different material formulations on performance. The study further conducted DS vs. GPT error analysis, quantifying the influence of different data sources on model predictions and providing a new approach for evaluating the reliability of AI-generated data. The feasibility of AI-generated data in material science was explored, and future optimization directions were proposed, including the development of GAN/VAE-based methods for generating high-quality material data to mitigate overfitting risks in GPT-generated data. Moreover, the integration of Graph Neural Networks (GNNs) and transformer architectures was suggested for advanced material property predictions, further expanding the role of AI in material science modeling. In summary, this study demonstrates that machine learning combined with AI-generated data can effectively predict key properties of low-temperature sealing materials and, for the first time, provides a comparative analysis of DS and GPT data applicability. By incorporating data augmentation, multi-model optimization, 3D visualization and error analysis, a novel approach for optimizing low-temperature sealing materials was proposed, offering a valuable reference for future data-driven material research.

## Figures and Tables

**Table 1:** Summary of common low-temperature sealing materials, their applications and limitations.

Material	Applications	Limitations
Silicone Rubber	Used in cryogenic storage, aerospace and medical devices [8]	Degrades under prolonged exposure to acidic environments
Fluoroelastomers	Suitable for aggressive chemical environments in automotive and aerospace [9]	High-cost limits large-scale application
Ethylene Propylene Diene Monomer (EPDM)	Widely used in PEM fuel cells and electrical insulation [10]	Lower chemical resistance compared to fluorinated materials
Polytetrafluoroethylene (PTFE)	Applied in chemical processing and corrosion-resistant gaskets [11]	Low mechanical strength often requires structural reinforcement with other materials

**Table 2:** Examples of actual experimental data.

Sample	Substrate	Compression Stress Relaxation (%)	Compression Set (%)	Rebound Resilience at Room Temperature (23 °C) (%)	Rebound Resilience at −40 °C (%)	Rebound Resilience at −30 °C (%)	Rebound Resilience at −20 °C (%)	Acid Resistance (Mass Change %)	Rebound Resilience After High Temperature/High Humidity Aging (90 °C/100%) (%)	Rebound Resilience After −40 °C Damp Heat Aging (%)	Surface Resistivity (Ω/cm^2^)	Volume Resistivity (Ω·cm)
EBT-1	EBT (K9330M)	10.36	8.591	48.83	12.20	19.90	27.14	−0.15%	49.04	10.92	1.95 × 10^10^	3.31 × 10^9^
EBT-2		11.1	8.938	51.77	9.04	20.77	28.24	−0.15%	52.41	10.11	3.42 × 10^11^	5.79 × 10^9^
EBT-3		11.34	8.938	53.93	9.73	26.17	31.87	−0.19%	53.39	8.94	7.79 × 10^13^	1.18 × 10^13^
EBT-4		13.07	14.196	55.52	10.35	25.28	30.71	−0.18%	54.91	10.57	8.09 × 10^13^	1.80 × 10^13^

**Table 3:** Examples of AI-predicted data.

Rubber	Softener	Crosslink Agent	Carbon Black	Calcium Carbonate	Rebound	Acid Resistance	log(Resistivity)
100	70	2.5	40	0	33	0.12	1.70 × 10^13^
100	70	3	40	5	32.8	0.09	1.68 × 10^13^
100	70	3	40	0	32.5	0.1	1.70 × 10^13^
100	70	3	45	0	32	0.12	1.50 × 10^13^
100	70	3	40	10	31.5	0.14	1.60 × 10^13^

**Table 4:** Comparison of valid prediction ranges (2).

Performance	Data Source	Softener	Carbon Black	Crosslinking Co-Agent	Calcium Carbonate
Low-Temperature Rebound rate	CHATGPT	60–70	40–47.5	2.75–3.25	0–10
	DEEPSEEK-V3	60–70	40–45	3–3.5	30–35
Volume Resistivity	CHATGPT	10–20	40–45	2.5–3.5	0–10
	DEEPSEEK-V3	50–70	40–45	3–3.5	40–50
Acid Resistance	CHATGPT	60–70	40–45	2.75–4.5	0–5
	DEEPSEEK-V3	60–70	45–50	2.5–3.5	0–10

## Data Availability

The original data presented in the study are openly available in zenodo at 10.5281/zenodo.15240233. The original contributions presented in this study are included in the article/Supplementary Material. Further inquiries can be directed to the corresponding author. The raw data supporting the conclusions of this article will be made available by the authors on request.

## Supplementary Materials

The following supporting information can be downloaded at: https://www.mdpi.com/article/10.3390/polym17091233/s1, Figure S1: Distributions of key performance metrics comparing experimental data and AI-generated datasets. (a) Rebound rate (%), (b) Log resistivity (log Ω·cm), and (c) Acid resistance (%).

## Abbreviations

The following abbreviations are used in this manuscript:

LLM	large language model
DS	DeepSeek-v3
GPT	ChatGpt
EPDM	Ethylene Propylene Diene Monomer
PTFE	Polytetrafluoroethylene
MLP	Multilayer Perceptron
LTSMD	Low-Temperature Sealing Material Database
GNNs	Graph Neural Networks
FEA	Finite Element Analysis
LASSO	Least Absolute Shrinkage and Selection Operator
SHAP	SHapley Additive exPlanations
PCA	Principal Component Analysis
SPIC	State Power Investment Corporation
EBT	EPDM-Based Trial Rubber (K9330M by Mitsui Chemicals)
PAO	Polyalphaolefin
Viscort#230	Commercial crosslinking co-agent supplied by Osaka Organic Chemical Industry Ltd.; enhances crosslink density and improves mechanical performance.
PERHEXA-C/PERCUMYL-D	Organic peroxide curing agents produced by NOF Corporation; used for thermal vulcanization of EPDM rubber.
SPHERON 5200	GPF-grade conductive carbon black manufactured by Cabot Japan K.K.; used to adjust electrical conductivity and mechanical reinforcement in rubber compounds.
phr	parts per hundred rubber
