# Supplementary material for: Low-Temperature Sealing Material Database and Optimization Prediction Based on AI and Machine Learning
Source: Polymers (Basel). 2025 Apr 30;17(9):1233. doi: 10.3390/polym17091233 (PMC12073874; doi:10.3390/polym17091233)
Supplement: Supplementary file 1 [file polymers-17-01233-s001.zip › Supplementary File/Supporting Information.pdf]

Supplementary Information – Section 2.4 Extended Details

1. Data Import and Preprocessing Details

The following details describe the data import and preprocessing pipeline used to construct the Low-Temperature Sealing Material Database (LTSMD).

Data Import Process:

- Traverse the material folders to locate .xlsx files.
- Read the Excel spreadsheets and clean invalid column names.
- Fill in empty cells using forward fill to avoid missing data.
- Store data in the SQLite database with automatically generated table names in the format: Material_Model_TableName (e.g., SyntheticRubber_EBT_Composition).

Data Cleaning Steps:

- Remove unnamed columns from Excel files.
- Apply forward filling (ffill) to ensure data completeness.
- Use `to_sql(..., if_exists="replace”) ` to avoid data duplication.

2. User Interface and Login Logic

Details of the database user authentication and interface components:

Login Interface:

- Users input username, password, and API key.
- Authenticated user info is saved locally.
- New user registration supported via POST requests to the server.
- Successful login grants access to the main interface.

API Key Management:

- API key must be input to unlock advanced functions.
- Stored securely during session-based operations.

3. Database Structure and Table Design

The LTSMD database organizes the data as follows:

Root directories include: Synthetic rubber, hot-melt adhesive, silicone rubber, and UV adhesives.

Each material category contains models (e.g., EBT, EPT, EPDM).

Each model has multiple Excel data files, categorized as:

- Material Composition
- Physical Properties
- Performance Testing

Example Table Naming Convention:

- SyntheticRubber_EBT_Composition
- Silicone_EPT_Properties

• UVAdhesive_Model1_Performance

4. Supplementary Figures

The following figures are referenced in the main text but detailed here for UI representation purposes.

Figure 1(a): Login interface showing username, password, and API key fields.

Figure 1(b): Main interface with options for material selection, query input, and result output.

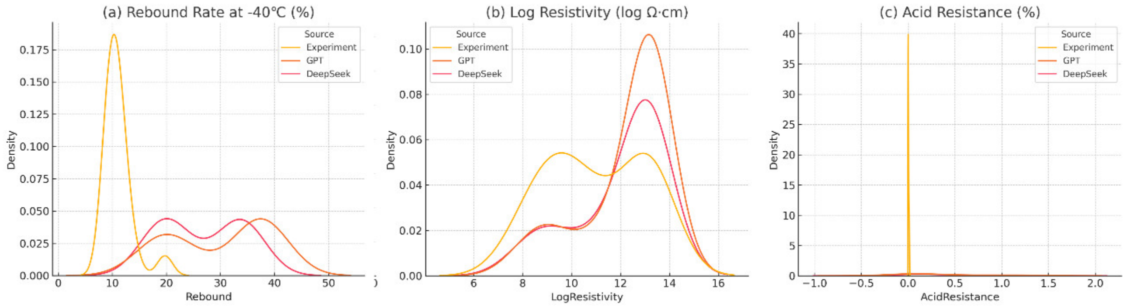

Figure S1. Distributions of key performance metrics comparing experimental data and AI-generated datasets. (a) Rebound rate (%), (b) Log resistivity (log Ω·cm), and (c) Acid resistance (%).

The statistical alignment between experimental, GPT-generated, and DeepSeek-generated data supports the realism and validity of synthetic data used in model training.
