# Supplementary figures and images for: Low-Temperature Sealing Material Database and Optimization Prediction Based on AI and Machine Learning
Source: Polymers (Basel). 2025 Apr 30;17(9):1233. doi: 10.3390/polym17091233 (PMC12073874; doi:10.3390/polym17091233)

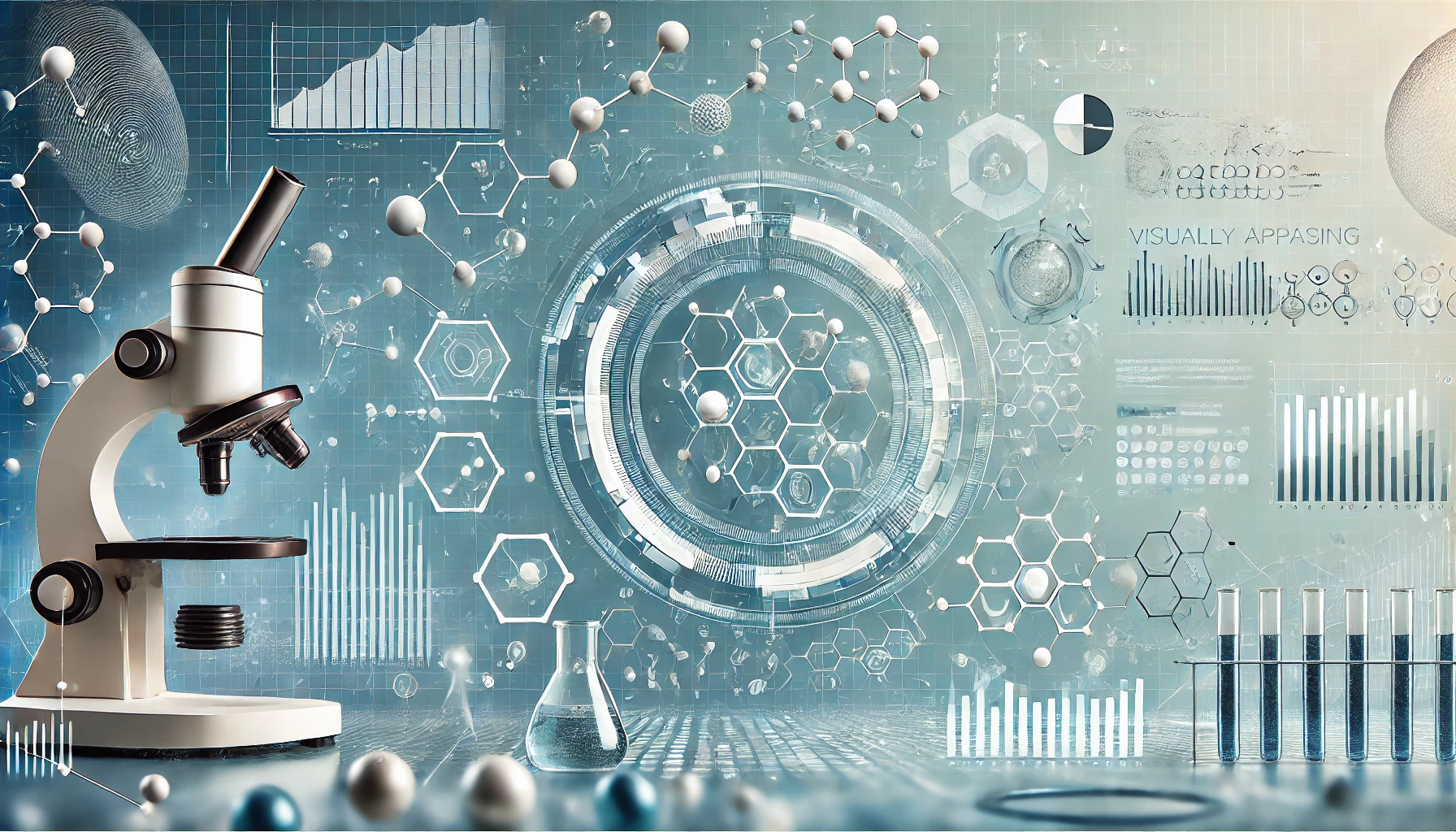

Supplement: Supplementary file 1 [file polymers-17-01233-s001.zip › Supplementary File/2.png]

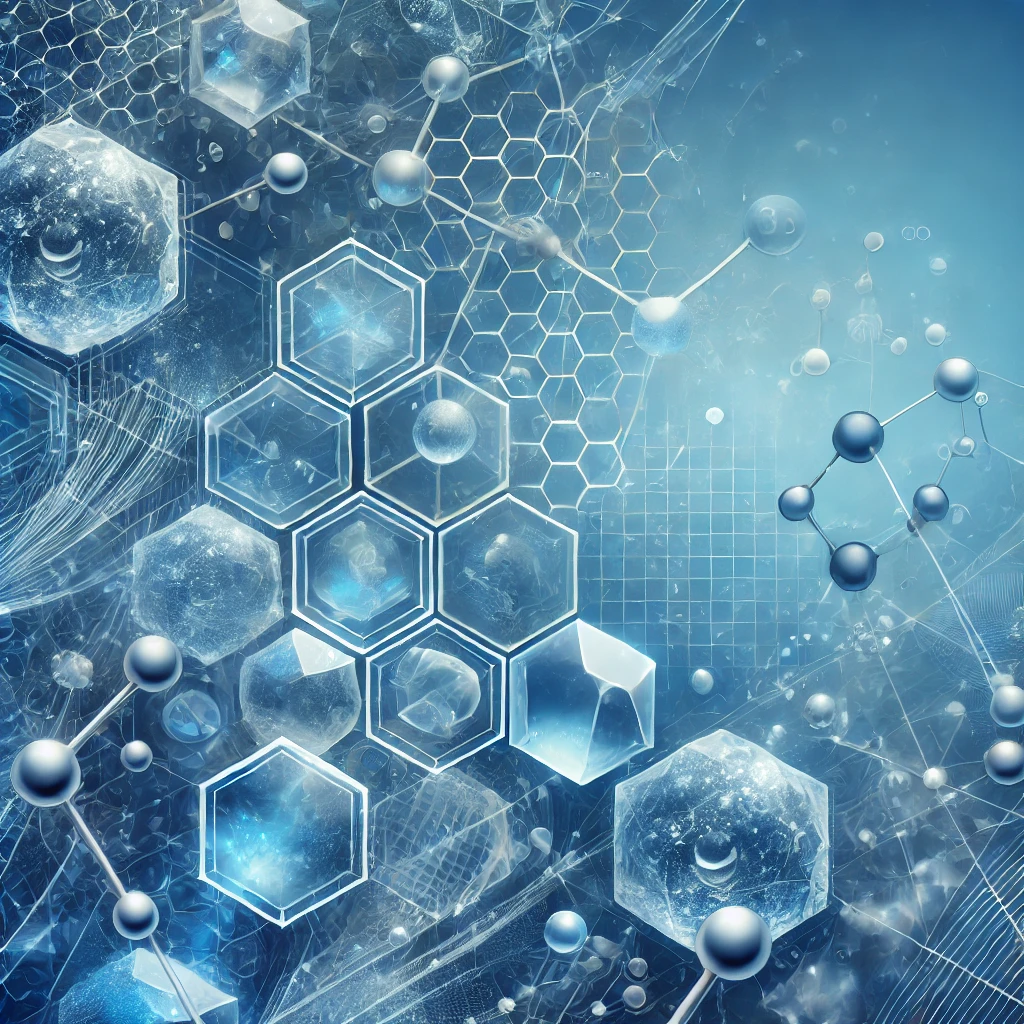

Supplement: Supplementary file 1 [file polymers-17-01233-s001.zip › Supplementary File/dl.png]
